# Controlling an Industrial Robot Using Stereo 3D Vision Systems with AI Elements

**DOI:** 10.3390/s25206402

**Published:** 2025-10-16

**Authors:** Jarosław Panasiuk

**Affiliations:** Faculty of Mechatronics, Armament and Aerospace, Military University of Technology, Kaliskiego 2 Street, 00-908 Warsaw, Poland; jaroslaw.panasiuk@wat.edu.pl; Tel.: +48-606835567

**Keywords:** industrial robot, vision system, system control, stereovision, FANUC robot, remote motion interface **(RMI)**

## Abstract

Robotization of production processes and the use of 3D vision systems are currently becoming more and more popular. It allows for more flexibility in the robotic process as well as expands the possibilities of process control, depending on changes in the parameters of the object, its pose, and changes in the process itself. Unfortunately, the use of standard solutions is limited to a relatively small space in which the robot’s vision system operates. The use of the latest solutions in the field of Artificial Intelligence **(AI)** and external vision systems, in combination with the closed structures of industrial robot control systems, provides advantages by enhancing the digital awareness of the environment of robotic systems. This article presents an example of solving the problem of low digital awareness of the environment of robotic systems resulting from the limited field of view of vision systems used in industrial robots, while maintaining high precision of the systems consisting of the combination of a 3D vision system using a stereovision camera and software with AI elements with the control system of an industrial robot from FANUC and an integrated Robot Vision **(iRVision)** system to maintain the positioning accuracy of the robot tool.

## 1. Introduction

The development of industrial robotics nowadays goes hand in hand with the dynamic progress of computer vision technologies. Traditional robotic systems, based on precisely defined trajectories and defined environments in which the positions and orientations of elements in space are defined and unchanging, are giving way to autonomous solutions capable of adapting in real time. The need to quickly change the assortment, production parameters, etc., forces the use of flexible system solutions. At the heart of this revolution are 3D vision systems, which enable robots to “see” and interpret their surroundings [1]. The article discusses the use of 3D vision technologies based on the integration of data from several sensors enriched with Artificial Intelligence **(AI)** elements in building operational awareness of industrial robots, as well as the benefits, challenges and future directions of development in this area [2].

The use of computer vision systems in industrial robotics applications has been common for many years [3,4]. It allows for the flexibility in industrial processes, enabling the acquisition of information about the pose of objects in the robot’s workspace using 2D and 3D cameras [5,6]. In most cases, vision systems integrated with robots perform functions related to determining the pose of objects in the workspace [7], and can also be used for trajectory correction, quality control and positioning of process points. In all these cases, the currently implemented vision systems are characterized by similar features, which, on one hand, are aimed at maintaining high precision of the robotic system [8], and on the other hand, they have the limitation of the wider use of the vision system to collect information from the robot’s environment and build digital operational awareness. This limitation is due to the relatively small angle of view of currently used industrial vision systems, as well as the relatively shallow depth of field at which the vision systems of industrial robots can work while maintaining high image quality parameters [9]. Most of the work and research in this area is focused on improving the accuracy of determining the pose of objects recorded by 3D vision systems [10,11], as well as the use of AI to provide flexibility in the object detection process [2,8,12].

The aforementioned digital operational awareness in the context of industrial robotics refers to the ability of a robotic system to perceive and understand its environment, particularly information about the presence and pose of relevant elements in a wider workspace, and to make decisions based on this information [13,14,15]. The next stage of consciousness acquisition is data analysis, understood as the processing of sensory information from the vision system and other sensors in real time (sensory fusion and extraction of information relevant to the robotic workstation). The development of control signals based on the collected and analyzed data allows for dynamic adjustment of the trajectory, speed and actions of the robot in response to the obtained knowledge about the location of objects in the workspace (considering the maintenance of all important process parameters and those responsible for safety at the station).

In the case of industrial robotic systems, stereoscopic cameras are the most commonly used, often enriched with additional systems generating structured light. The combination of these two elements allows for high precision in determining the pose of objects recorded by such a 3D vision system. An example of this type of solution is a 3D vision system offered by companies such as FANUC, Cognex or Keyence. Tests carried out in the laboratory show that with relatively small pitch and yaw angles of objects of +-10 degrees, angle measurement errors should not exceed tenths of a degree. This value is sufficient to effectively remove elements from bins or other containers containing many elements of a given type.

The use of 3D computer vision systems in robotic stations, as already mentioned, brings a number of benefits. In particular, attention should be paid to increasing process flexibility, thanks to which robots can work in environments with high variability, which eliminates the need for costly preparation of positioning fixtures and static programming of trajectories. 3D vision systems can also have a significant impact on improving safety by integrating with advanced threat detection systems, minimizing the risk of accidents. Unfortunately, in this area, the use of computer vision systems must be in accordance with applicable safety standards and thus must meet strict requirements for reliability and responsiveness. The use of 3D systems also allows for precise movement planning, bypassing potential obstacles and, thus, shortening the production cycle time.

Despite the indicated potential, the implementation of 3D vision systems is associated with certain challenges. The first is the relatively high costs, which include both hardware and software, as well as integration and configuration costs, which each time are individual to a given process. Another challenge is the complexity of the data processed in 3D vision systems [16]. The analysis of large amounts of information requires significant computing power, which is often a limitation for closed control systems of industrial robots. However, there are already solutions in this area, such as the FANUC integrated Personal Computer **(iPC)**, which is an additional computing unit responsible for the implementation of particularly complex algorithms. When using both 2D and 3D vision systems, it is important to be aware of sensitivity to external conditions: lighting, dust and other factors can affect the quality of the data, which means that in many cases, the possibility of their use is limited or requires additional investments to eliminate, for example, contamination [5].

The solution presented in the article, consisting of building digital operational awareness of the robot’s environment using various vision systems, as well as the results presented, can be the starting point for further work, allowing for the development of more flexible algorithms for the operation of robotic stations. This is particularly important due to the fact that the solutions currently being introduced by robot manufacturers include elements that enable much greater integration of external systems with closed robot control systems. An example of this is FANUC, which enables the use of Python 3.10 code in the latest R50iA controllers. In addition, a dedicated iPC computing unit is available, whose main task is to take over more demanding computing tasks from the robot controller, especially in the field of data processing from vision systems. All this means that we are currently at a moment of significant change in the philosophy of programming industrial robotic systems. The latest industrial robot controllers seem to be ready to implement popular AI algorithms [2,17], both in terms of image processing and the control itself, which will be more efficient. It should also be borne in mind that the coming years, based on the above-mentioned emerging hardware solutions, will bring innovations, such as broadly understood integration with AI, thanks to which it will be possible to significantly improve the ability to interpret video data. The first solutions of this type are already available, such as AI Error Proofing by FANUC. However, their use is currently limited to quality control operations. Another innovation will be the miniaturization of sensors and improved performance.

The developed solution presents a new approach to building spatial awareness of the robot system. Previous computer vision systems integrated in robotic systems used solutions focused on a small workspace. Thanks to the use of an AI algorithm to detect elements of different classes, which was not previously possible with industrial solutions, it was possible to make robotic process solutions more flexible. The use of an additional communication interface, an external computer and one or two 3D cameras with a wide field of view creates virtually unlimited possibilities to increase the functionality of the robotic station. 3D cameras observing the workspace provide full information about what is happening on the robotic station, and the use of an external computing unit does not interfere with the safety and stability of the robotic process. Integration of systems operating on a wide and narrow field of view provides the best of both worlds. The presented solution has been verified in laboratory conditions, allowing for obtaining results in accordance with the expectations (detection of the position of objects in the robot’s workspace, belonging to a specific class, using an AI algorithm and precise determination of the object’s pose in order to carry out further technological processes).

## 2. Related Work

Vision systems based on 2D image analysis have been the foundation of automatic inspection solutions for years and increasingly support robotic work. For example, these systems are used for object location, tracking, and verification. Their relative simplicity of implementation and lower hardware costs were the factors that popularized this class of methods in industrial settings. The key advantage of 2D computer vision solutions is the maturity of the available algorithms and a large resource of programming libraries, as well as ready-made tools that can be used by engineers and scientists. Therefore, in recent years, especially with the development of deep learning algorithms and the growing availability of sensors recording scene depth information, there has been an intense increase in interest in 3D computer vision systems [18]. These scene depth data measuring solutions enable more precise reconstruction of the shape of the object and more reliable localization in the environment, which translates into the ability of robots to perform complex assembly, sorting or palletizing tasks. A significant advantage is also the ability to decompose the scene and distinguish partially obscured objects, which can be unreliable in classic 2D systems.

In the latest literature on computer vision systems and AI elements on robotic stations, the following main research directions and open issues are evident:

- Deep learning methods are integrated into vision-based robotic systems to improve object detection and classification performance. The introduction of AI to the analysis of images and point clouds has made it possible to transfer tasks related to segmentation or estimation of the position of objects from classic image processing algorithms to deep models. In particular, the YOLO algorithms and their subsequent variants (YOLOv4, YOLOv5, YOLOX, YOLOv7, YOLOv8) demonstrate high computational performance and are successfully used in vision systems at robotic stations [19,20,21].
- The development of 3D algorithms based on data from stereo cameras and depth sensors has allowed for the design of methods that combine the analysis of 2D information (texture, color) with 3D information (depth) [22,23,24]. Recent work focuses on optimizing these approaches for precision, computational speed and reliability, which is crucial in industrial applications. In these cases, speed and reliability translate directly into the efficiency of the production line, as possible errors can cause problems, even leading to damage to elements of production stations, especially in the event of collisions between machines and equipment.
- Adaptive methods of robot control based on current information are being developed, while maintaining procedures that ensure robotic-process safety [25];
- Real-time location and tracking of objects is extremely important for the robotization of processes in which an object can move (e.g., on a conveyor belt) or when a robotic arm must dynamically respond to changing conditions (e.g., in assembly in motion). Therefore, lightweight and optimized neural network architectures are being developed, allowing for the implementation of 3D detection with high throughput (e.g., several dozen frames per second) while maintaining high reliability [26,27];
- Calibration and fusion of sensors are among the most difficult aspects of the implementation of 3D systems. It is important to calibrate and synchronize multiple sensors so that information from different sources is combined consistently and made suitable for further processing. Intensive research is being carried out on methods of automatic and semiautomatic calibration using spatial markers or so-called structural features. The aim of this work is to reduce the impact of measurement errors on the efficiency of detection and location of objects in the robot’s coordinate system [28,29];
- Computer vision systems are being adapted to industrial conditions, which should be understood as resistance to difficult lighting conditions, dust or vibrations. New research directions are exploring adaptive solutions in which the vision model can automatically tune to changing conditions, minimizing the decrease in detection quality. Few-shot learning methods or generating artificial data sets (data augmentation in 3D space) are also considered [30,31,32];
- Maintaining a favorable balance of complexity and efficiency versus cost. Deploying high-quality robotic vision systems can be expensive, especially for small and medium-sized businesses. Simplifying and reducing the cost of these systems while maintaining their high functionality remains a key area of focus.

In industrial environments, solutions that are highly reliable and versatile are the most popular, adapting to changing product ranges and environmental conditions while maintaining adequate image processing bandwidth. This development will most likely intensify with technological progress in the area of 3D sensors and further optimizations of neural network architectures, in particular those related to YOLO, which is already confirmed by numerous publications from recent years. Unfortunately, the technological complexity and its cost in the use of computer vision systems in robotic systems are increasing. Therefore, it is advisable to develop a solution that will allow for a significant increase in the capabilities of existing robotic station solutions, with a relatively low implementation cost.

Available articles formulating the principles of cooperation of the system of cameras mounted on the robot arm and outside the arm were presented in the literature as early as 2000 [33]. Subsequent papers presented the issues of data fusion from multiple cameras, the selection of their parameters and redundancy management [34]. In subsequent works by the same authors, we can find a description of the classic implementation of the industrial hybrid Eye-to-Hand (EtoH)/Eye-in-Hand (EinH) [35]. Further work of the researchers was aimed at developing hybrid system solutions, taking into account switching the signal source. The article Hybrid Multi-camera Visual Servoing to Moving Target [36] describes a system with four RGB-D EtoH + stereo EinH cameras and a supervisor switching the source by distance/occlusion. Similar topics were maintained in the works [37,38], presenting hybrid image-based visual servoing (IBVS) with switching to the tip camera when the target appears in the FOV and describing the following strategy: global positioning of EtoH → local attachment of EinH.

In this paper, the next step in research on the use of hybrid vision systems in robotic applications, particularly in industrial robotics, is presented. Based on the above-mentioned work and research, as well as market needs, a solution was proposed and tested that would allow for the construction of digital spatial awareness using cameras with complementary functionalities, taking into account the latest solutions in the field of object recognition using AI algorithms. The effectiveness of the proposed solution ensures its practical usability, as well as the possibility of further expansion, although with functions related to safety supervision at robotic workstations.

## 3. Materials and Methods

Modern industrial robotics strives to increase the autonomy and adaptability of robots and entire robotic and production stations. A key element of this process, as already mentioned in the introduction, is an advanced perception of the environment, enabling robot systems to identify objects, analyze the workspace, and detect obstacles and potential threats to both the robot and the process using mathematical models [39]. Ultimately, it is important to detect situations in which humans are at risk [40], which may be caused by a control system or human error [41]. In this context, mathematical models play an important role in providing formal tools for the description and analysis of perceptual processes.

Mathematical models are also the foundation of the description of perceptual processes of robots and robotic stations, because they allow for the formalization of complex phenomena, which enables their analysis, simulation and implementation in control systems, enabling the optimization of industrial processes.

Examples of the use of mathematical models of robots already at the stage of designing robotic stations are virtual environments, such as RobotStudio [42] by ABB or Roboguide [42] by FANUC. These environments, using models of robot kinematics and dynamics, allow the construction of digital twins of entire robotic stations. In the context of environmental perception, several key models are distinguished:

- Kinematic and dynamic models record the robot’s movement and interactions with the environment, taking into account the forces and moments acting on the manipulator. They are essential for trajectory planning and cycle time calculation, thus allowing optimization of the robotic process and its parameters to achieve the best possible station performance.
- Geometric models are used to represent the shape and position of objects in the station’s workspace. They enable the creation of environmental maps and the identification of objects and potential obstacles. As a result, potential collisions at the robotic station (in the digital twin) can be detected before the physical station is created.
- Probabilistic models, used under sensory uncertainty, allow for consideration of measurement errors and incomplete data, especially for aspects of phenomena that are random in the real world. An example is the Kalman filter, used to estimate system state from noisy sensory data; another is an object position generator. In the case of environments such as Roboguide, probabilistic models allow the random generation of object arrangements to verify the correct operation of 2D and 3D computer vision systems. This allows verification of correct vision system operation for a robotic station already at the design stage.

In the case of real-world (physical) robotic stations, it is necessary to obtain information from the robot’s environment to react to differences between the virtual model’s ideal state and the real state (position of objects in the robot’s space, presence of people, etc.). In this context, object recognition at real industrial workstations is based on the analysis of sensory data, mainly 2D and 3D images [43]. It can be carried out by external vision systems from companies such as Cognex or Keyence, which communicate with the robot via communication protocols or by systems fully integrated with the robot controller, as in the FANUC integrated robot vision **(iRVision)** system [44]. The standard solutions currently available in industrial robot systems usually use advanced image analysis and processing algorithms. Mathematical models, such as geometric transformations, are crucial in the process of identifying and locating objects. A new trend is the use of machine learning. An example of an application is the use of convolutional neural networks **(CNNs)** [45] to classify objects based on features extracted from images. These algorithms learn data representation, which allows effective object recognition even in complex scenes [46]. The study design, which verifies the possibility of increasing the robot’s digital spatial awareness, envisions integrating a robot with external information sources into a single system in which a PC processes data from a stereovision camera. When designing the study, the working area was divided into a detection/identification area and a precise positioning space of the tool in relation to the detected object. This is due to the different accuracies of the vision systems operating in the detection and precise positioning spaces. To verify the algorithm’s correctness, dedicated software was created to calibrate coordinate systems and convert data from the stereovision camera’s coordinate system to the robot’s.

### 3.1. Elements of the Test Stand

**Figure 1:**
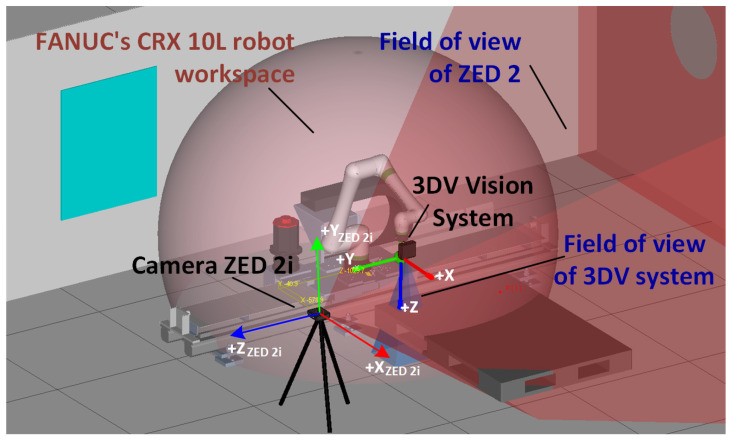
Roboguide test bench model (Digital Twin).

Constructing the test bench (Figure 1) to verify the proposed concept of obtaining data using a wide-angle stereovision camera required defining the station’s components and their roles to achieve the intended functionality [47].

It was assumed that the source of rough data about the position of objects in the robot’s workspace would be the ZED2i stereovision camera from Stereolabs, placed in a fixed position in relation to the industrial robot. The camera will be connected to an external PC with proprietary software using Stereolabs libraries 4.0. On the 2D image obtained from the stereovision camera, the object will be located using the YOLOv8 algorithm, applying a set of neural network weights, which will be obtained in the proprietary learning process based on the collected training data. The 2D position data obtained in the object detection process, in combination with the depth map data, will be converted to 3D coordinates. The spatial position data of the detected object will be converted to the robot’s coordinate system and then transmitted to the robot controller to align the robot’s vision axis directly over the detected object for precise positioning and further robotic process operations according to data from FANUC iRVision.

Building the station in accordance with the adopted assumptions (Integration of the ZED2i camera and FANUC iRVision 3D Area Sensor) required the following:

- Precise calibration of the camera and determination of a common coordinate system;
- Close synchronization and division of tasks in the control algorithm;
- Development of software integrating both systems and additional software allowing for the implementation of configuration and verification of the correctness of measurements.

The station where the commissioning tests and research were carried out consists of a FANUC CRX-10iA/L industrial robot with the R30iB Plus controller, the integrated iRVision system, and a hardware module in the form of a 3DV/400 structured-light stereovision camera. In order to ensure communication with an external computer system, the robot controller software has been expanded to an RMI module. This solution enables control over the manipulator’s motion from external devices and allows reading key robot-status parameters. In this setup, RMI establishes communication and sends commands to move the manipulator tool toward the detected object’s coordinates, considering the position elevation (height offset) so the camera is positioned directly above the object. This is designed to ensure that the 3DV video system captures the object within its field of view and at a distance that ensures correct iRVision system operation. The keypad control data from the external computer system are computed from Stereolabs ZED 2i camera measurements and sent to the controller via FANUC RMI library commands.

### 3.2. ZED2i Stereolabs Camera and Yolo Object Detection Algorithm

The ZED 2i by Stereolabs is part of a solution that includes components such as the camera itself (available in five versions with different parameters), proprietary software provided with libraries that allow for camera configuration and reading data from the camera, a dedicated computing unit using NVIDIA processors and dedicated cables that enable connecting individual hardware elements. Due to the fact that both parts of the software dedicated to the ZED 2i camera and the object detection algorithm (YOLO) [48] have been combined in one application, operating on the same computing unit, they are discussed here together.

The main reasons for choosing ZED 2i are the relatively low price of the equipment and the parameters that provide a large depth range of 3D image, as well as support in the form of the manufacturer’s libraries (Stereolabs). Libraries are being developed all the time and, as pointed out in the article, a single camera has a dead zone caused by occlusion from the robot’s manipulator. Stereolabs has released libraries for the real-time integration of data from two ZED 2i cameras; this will be the subject of a future article. With regard to using the YOLOv8 algorithm, the choice is primarily due to the ease of implementation and the ability to easily and relatively quickly train the network. Due to the practical nature of the solution presented in the article, it is also important that the developed solution can be easily adapted to new products by replacing the trained weight set.

#### 3.2.1. ZED 2i Stereolabs Camera

The Stereolabs ZED 2i (Table 1) is a stereo camera that uses two 4 MP CMOS sensors to capture images with a resolution of up to 4416 × 1242 pixels at 100 frames per second. It features a 120° field of view, providing a wide perception of the environment. In addition, the camera is equipped with a set of sensors, such as a built-in IMU, barometer, and magnetometer; these collect inertial, altitude and magnetic data, facilitating integration of the camera with other coordinate systems [49]. The ZED 2i supports depth perception, motion tracking, and spatial AI to support the development of advanced systems that understand their environment [50]. 

When configuring the ZED2i camera using the libraries provided by Stereolabs, many parameters can be set. One of the most important aspects of the test stand is the configuration of the coordinate system in which the position of detected objects are measured.

The calculation of the depth of Z in a stereovision system is based on the parallax difference d as follows:

(1) $$d=x_{L}-x_{R}$$

- ***x_L_***—left camera position;
- ***x_R_***—right camera position.

If the corresponding points in the left and right images are misaligned (e.g., due to noise), the parallax error results in depth error.

The depth error ΔZ, can be calculated from the following relation:

(2) $$∆Z=\frac{f\cdot B}{{\left(d+∆d\right)}^{2}}\cdot ∆d,$$

- **f**—focal length of the camera;
- **Δd**—correspondence point matching error;
- **B**—stereovision base (distance between cameras).

Depth accuracy decreases with the square of distance Z; stereo depth accuracy ranges from 1% of the distance at short range to 9% at long range. Depth accuracy can also be affected by outlier measurements on homogeneous or textured surfaces, such as white walls, green screens, and mirror areas. These surfaces usually generate temporal instability in depth measurements.

According to Stereolabs, the manufacturer of the ZED 2i, and provider of the libraries used, the configuration that provides the best depth accuracy places the camera 30 cm to 1 m from the scene.

Calibration errors in the stereovision system can also cause incorrect internal and external camera arrays, which affect the accuracy of the 2D to 3D coordinate transformation.

If we assume that

- **K_L_**, **K_R_**—inner matrices of the left and right views;
- **R**, **t**—rotation matrix and translation vector between cameras.

and if the matrices K_L_, K_R_, R, and t contain calibration errors (ΔK_L_, ΔK_R_, ΔR, Δt), the resulting coordinates of the object (X, Y, Z) will be affected by the error as follows:

(3) $$∆P_{c}=∆K\cdot P_{i}+\left(∆R\cdot P_{i}+∆t\right),$$

- **P_i_**—the actual coordinates of the object in space,
- **P_c_**—detected coordinates.

Although the manufacturer allows calibration of ZED 2i cameras, it is not recommended. Therefore, it can be assumed that we have no direct influence on this type of error; it is a factory error related to a specific camera unit. Unless there has been physical interference with the camera structure, it is not advisable to calibrate such a camera.

When analyzing the overall issue of the use of an external stereovision camera in the robot system, it is also necessary to take into account errors related to the transformation between the camera (C) and the global system associated with the robot (W). Converting coordinates from a camera system to a global system may be subject to errors in the T_CW_ transformation matrix. If the T_CW_ contains errors in rotation (ΔR_CW_) or translation (Δt_CW_), the calculated position of the object in the global system (X_w_, Y_w_, Z_w_) will be affected by the error as follows:

(4) $$∆P_{\omega }=T_{CW}\cdot ∆P_{c}+∆{T_{CW}\cdot P}_{c}.$$

The introduction of noise and calculation errors in turn leads to the accumulation of errors in the final result [8]. If we assume that η_X_, η_Y_, and η_Z_ denote the noise in the input data, then the final error of the position in 3D space is as follows:

(5) $$∆X_{final}=∆X+\eta_{X},\;\;\;\;∆Y_{final}=∆Y+\eta_{Y},\;\;\;\;∆Z_{final}=∆Z+\eta_{Z}.$$

#### 3.2.2. Yolo Object Detection Algorithm

Object detection and position calculation on the 2D image were carried out using the YOLOv8 algorithm. As newer and more effective solutions emerged, the algorithm was retrained, this time with the YOLOv8 version. During training, similar results were obtained, but with a significant reduction in the network structure. For the YOLOv5 algorithm the structure in the “small” size was used, and in the case of the YOLOv8 network, the “nano” size was used.

Both YOLOv5 and YOLOv8 (Table 2) are powerful object detection models from Ul-tralytics. YOLOv5 remains a robust choice for applications prioritizing speed and effi-ciency, especially in resource-limited scenarios. YOLOv8, being the newer model, offers improved accuracy and broader task versatility, making it suitable for more demanding applications and research-oriented projects. The choice between YOLOv5 and YOLOv8 depends on the specific requirements of the project, balancing factors like accuracy, speed, and available computational resources. Both YOLOv8 and YOLOv5 are fast object detec-tion models, capable of processing images in real time. However, YOLOv8 is faster than YOLOv5, making it a better choice for applications that require real-time object detection.

**Figure 2:**
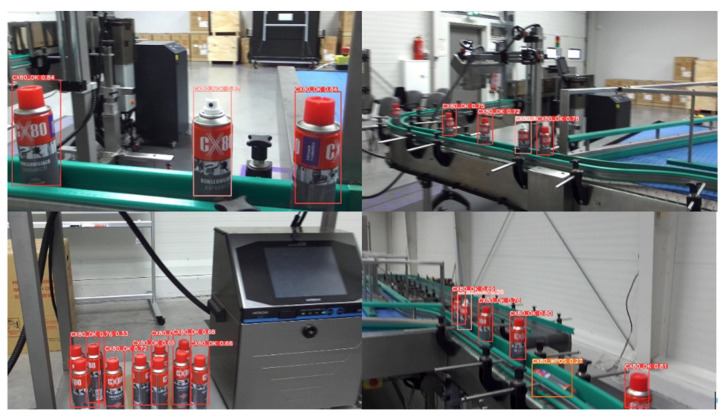
Sample images from the learning collection showing CX80 objects in categories: CX80_OK and CX80_NOK.

Object detection by the YOLO algorithm is carried out on the basis of a bounding box with a specific center (x_c_, y_c_) (Figure 2). If the rectangle is not precisely positioned over the object, the detected coordinates may deviate from the actual coordinates in the image. This is especially relevant when the object plane is not parallel to the image plane.

Error detection in the 2D image is as follows:

(6) $$∆x=x_{c}^{pred}-x_{c}^{real};∆y=y_{c}^{pred}-y_{c}^{real}.$$

This error propagates to an error in the 3D space, because 3D coordinates are a function of x_c_ i y_c_, as follows:

(7) $$∆X=\frac{∆x\cdot Z}{f};∆Y=\frac{∆y\cdot Z}{f},$$

- **Z**—depth (distance of the object from the camera),
- **f**—focal length of the camera.

**Figure 3:**
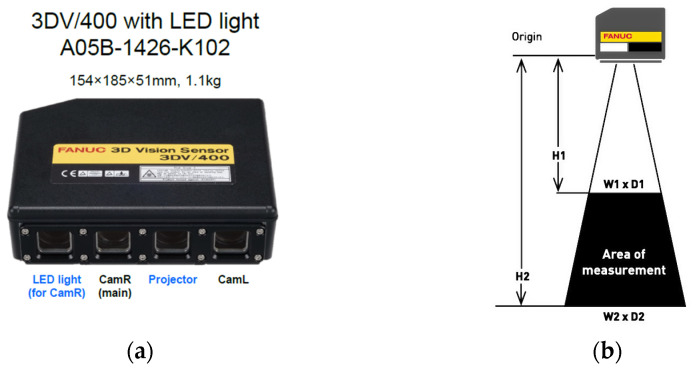
FANUC 3DV system (**a**) elements of the camera, and (**b**) area of measurement.

In the case of the test bench, the error could have been significant because, as can be seen in the attached sample photos from the training set (Figure 3), the centers of the bounding boxes did not coincide with the center of the object. This was largely due to the number of photos used to train the YOLO algorithm, in which the lower part of the CX80 container was obscured. Errors can come from multiple sources, such as detection by YOLO, stereovision depth calculation, camera system calibration, and transformations between coordinate systems. Precise mathematical modeling and analysis of these errors are crucial to improve the accuracy of the system and reduce deviations from the actual position of the object.

In the case of the ZED2i camera assembly and the YOLOv8 algorithm, the total error of the determined position P_err_ = (X_err_, Y_err_, Z_err_) in the 3D space results from the sum of the above errors as follows:

(8) $$P_{err}=P_{det}+P_{depth}+P_{calib}+P_{transform}+\eta ,$$

- **P_det_**—detection error by YOLO (object position vs. bounding box position),
- **P_depth_**—depth error in stereovision (influence of lighting conditions and surface type),
- **P_calib_**—camera calibration error (in the case of ZED2i, factory calibration),
- **P_transform_**—error of transformations of coordinate systems,
- **η**—system noise.

### 3.3. CRX-10iA/L Fanuc Robot with iRVision 3DV

The CRX-10iA/L collaborative robot, which is a part of FANUC’s entire line of collaborative robots, was chosen as the robot for the study. It is fully compliant with the safety standard. The choice of this particular robot was supported because it was equipped with the necessary data exchange interface and the integrated iRVision 3DV vision system. FANUC is one of the few companies producing industrial robots to offer three types of 3D vision solutions. These include the following systems:

- 3D Laser **(3DL)**;
- 3D Area Sensor **(3DAS)**;
- 3D Vision **(3DV)**.

The first of these, to determine the pose of objects in space, uses information about the position of the cross on the object projected by the laser projector. This allows the determination of the pose of objects of significant size, for which the crosshair arrangement can be helpful for camera data-processing algorithms. The system itself is equipped with a single 2D camera and a laser projector.

The 3D Area Sensor system uses a system of two cameras and a structured light projector. This system is used to locate and determine the orientation of many elements placed randomly in various types of bins or other collective containers. With this system, a sequence of images of a binary pattern is displayed on the surface to be analyzed, and based on eight recorded images, the system searches for and determines the pose of the learned elements [51]. The 3DV (Table 3) (Figure 3) sensor uses integrated blue LEDs as a light source to project the pattern. The device emits a single light pattern per object, which enables fast and accurate 3D data acquisition.

This solution differs from the 3D Area Sensor system, in which the projector emits several patterns on the analyzed surface and calculates their pose based on the analysis of the distribution of patterns on the surface of the elements.

The iRVision 3DV sensor technology, which uses structured-light projection to create 3D maps, allows parts to be recognized and selected based on the evaluation of the integrated part manager, indicating the fastest picking option The system is fully integrated into the robot controller, allowing the robot to be controlled directly based on vision data. It is dedicated to tasks such as depalletizing, bin picking, and other material-handling applications, even in difficult lighting conditions or with objects that have complex surfaces.

Errors in structured light systems, such as FANUC and RVision 3DV, are due to limitations in calibration, triangulation, pattern detection and orientation. A mathematical model of these errors allows them to be analyzed and reduced in practical applications.

**Figure 4:**
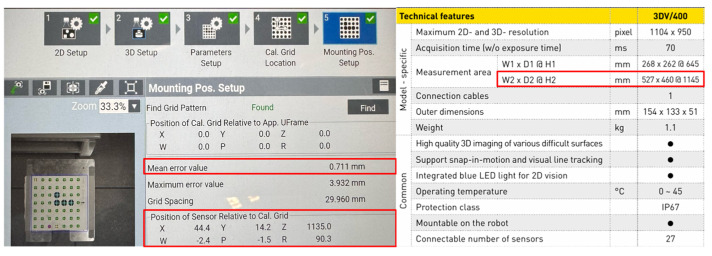
View of the calibration window of the 3DV iRVision vision system in relation to the catalog parameters of the 3DV/400 version used.

3D systems based on structured light, such as FANUC iRVision 3DV, use the projection of light patterns onto an object and the analysis of the distortion of patterns in the camera image to reconstruct spatial geometry. This process is prone to various errors due to system parameters, environmental conditions, and facility characteristics. The total error in PC position and R orientation for a 3DV system is due to the sum of errors from each error source, such as calibration error, triangulation error, and detection error (Figure 4).

(9) $$∆P_{total}=∆P_{calib}+∆P_{triang}+∆P_{detect}$$

Unfortunately, due to FANUC’s closed and patented design, the user can influence only the calibration of the vision system using the procedures included in the iRVision system.

The accuracy of pose determination by the 3DV vision system depends on the object’s position in relation to the axis of the optical system. In the center of the image, the error is the smallest, while on the periphery, it is the largest. During vision system configuration, it is possible to determine the maximum and average error for a given calibration grid. In the analyzed case, a manufacturer-provided calibration mesh with a spacing of 30 mm between the mesh points was used. The maximum error was 3.932 mm, while the average error was 0.711 mm. Thus, considering the repeatability specified by the manufacturer of the robot (0.05 mm), the maximum tool positioning error is no more than 4 mm. It should be noted that another source of error may be a TCP configuration error, which is used for both the configuration of the vision system (indicating the beginning of the grid coordinate system as well as determining its orientation) and the positioning of the tool itself.

### 3.4. Communication PC-Fanuc Robot with Remote Motion Interface (RMI)

**Figure 5:**
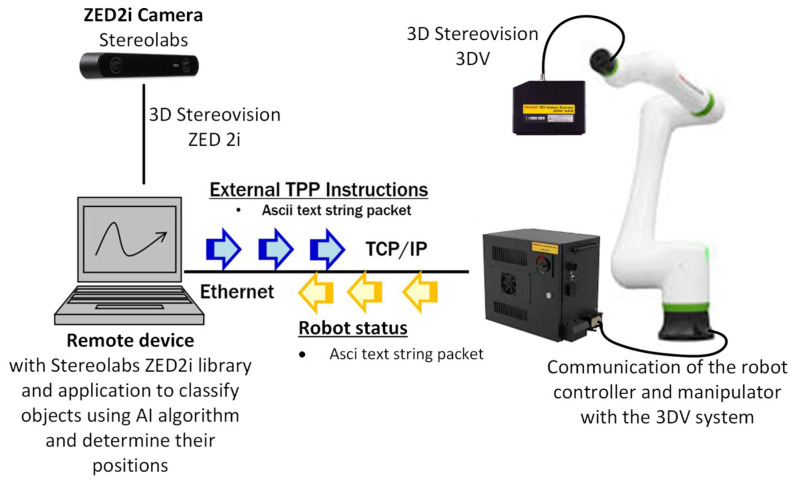
Diagram of the test bench consisting of a FANUC CRX robot with a 3DV/400 vision system and an RMI software (R912) package [53], an external computer with software (using Stereolabs libraries) and a ZED2i camera.

The use of data from the ZED 2i camera in the robot system requires processing and sending data from a computer running software that analyzes the data from the camera (3D image analysis, Yolov8) (Figure 5). To do this, a dedicated communication protocol [52] authorized by the robot manufacturer is necessary. Additionally, a library is required to send commands to control the robot and to call instructions that provide access to the robot’s system of functional tools data. For the test bench, it was necessary to send keypad movement instructions to align the 3DV camera axis directly above the position indicated by the ZED 2i camera and to start the iRVision system vision process.

**Figure 6:**
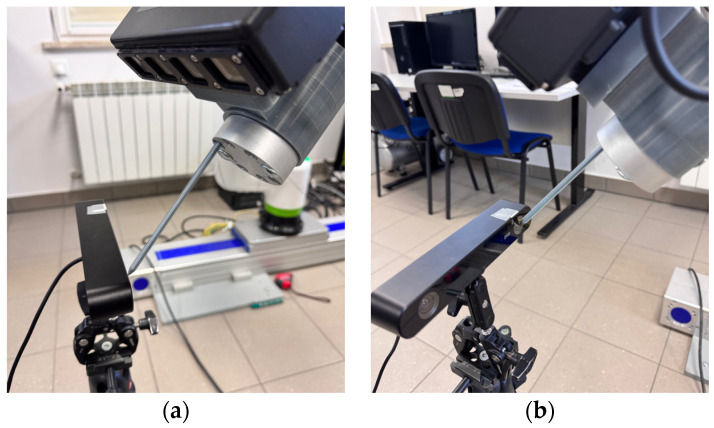
Configure the UserFrame at the ZED2i camera origin. (**a**) The direction of the *X*-axis, (**b**) the center of the UserFrame.

For the ZED2i camera and robot systems to operate in the same coordinate system, the ZED 2 had to be calibrated against the FANUC robot coordinate system. To do this, the robot’s UserFrame is configured at the origin of the ZED2i camera’s coordinate system (Figure 6). The transformation of data from ZED2i to FANUC iRVision 3DV coordinate system is as follows:

(10) $${P_{FANUC}=T}_{ZED-FANUC}\cdot P_{ZED},$$

- **P_ZED_**—ZED2i data;
- **T_ZED-FANUC_**—a transformation matrix between coordinate systems.

**Figure 7:**
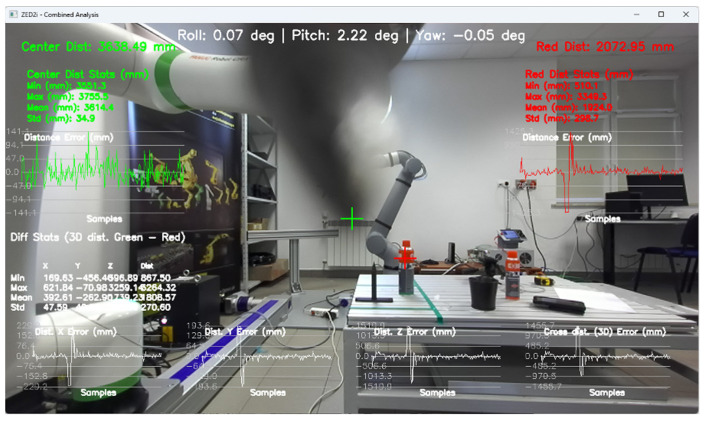
The center of the UserFrame coincides with the origin of the ZED2i camera’s coordinate system.

To configure the user coordinate system, which is linked to the ZED2i camera’s coordinate system, proprietary software was developed to record the camera’s tilt and yaw angles, which are visible at the top of the image (Figure 7).

The application also allows for measuring the distance to the central point of the image, based on the depth map, and to the point indicated by the user. Such a solution allows for the initial verification of the correctness of the distance measurement based on the depth map.

**Figure 8:**
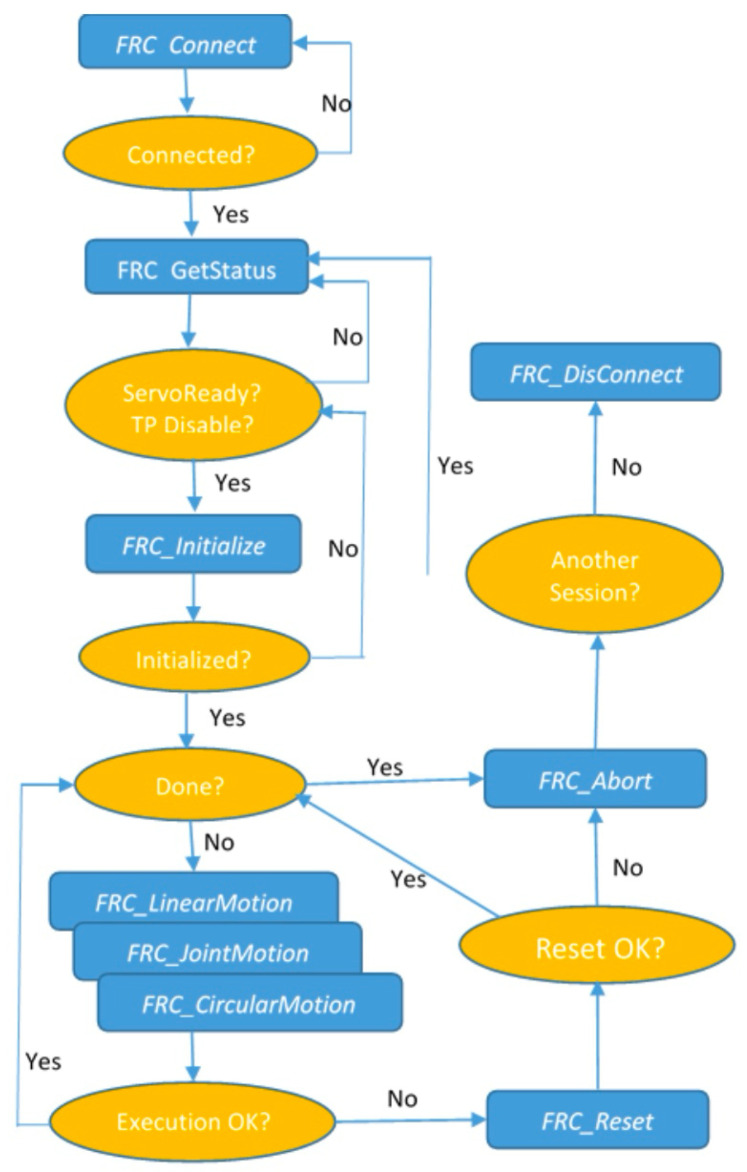
Remote device control flow chart [47].

After reading the data from the ZED2i about the position of the detected object in the camera coordinate system compatible with the coordinate system defined in the robot, the computer should communicate with the industrial robot using the RMI package. Commu-nication follows a scheme (Figure 8) that includes establishing a connection, initializing parameters, configuring the system, issuing instructions for movement, and calling up ro-bot programs. First, the address and port parameters must be declared according to the robot controller settings. Next, the connection process is carried out. The remote device sends the data packet to a specific robot controller port (port number 16001) to start a re-mote motion interface session. The robot controller returns the packet with integerValue = 0 to indicate that the connection is established.

If the current state of the controller is uncertain, the ***FRC_GetStatus*** packet can be sent to obtain the controller status. If the initialization command is executed successfully, the return integer value is 0.

The remote device sends this packet to the controller when it wants to start adding instructions to the TP program. Before sending this command to the controller, the robot controller must be in the following state:

- The teach pendant is disabled and the controller is in AUTO mode;
- The controller is ready to run, i.e., there are no other errors;
- The selected TP program is not RMI_MOVE.

In the next step, following the principles of programming industrial robots, the active user coordinate system **(UFrame)** and user tools system **(UTool)** in which the robot movement will be executed are set.

With the coordinate systems configured, the robot can be sent to a position by providing the object coordinates calculated from the ZED 2i data as parameters. For the “Y” position, an overhead must be added so that the camera is located above the object’s position to ensure the correct operation of the iRVision 3DV system.

Once the robot is in the designated position, the TP program can be called, in which the vision process is triggered. This is one of the variants of the control solution based on data transmitted via RMI. It is also possible to directly call up a vision process running in the background and implement further control based on the data read from the robot’s vision register. Once the program is finished, which can obviously be more complex, the connection must be terminated.

Combining the ZED 2 camera from Stereolabs with the FANUC iRVision 3D Area Sensor system allows taking advantage of both technologies. The ZED 2 camera provides wide ambient perception and dynamic tracking, while the FANUC iRVision 3D Area Sensor provides a precise pose of objects in the working space.

### 3.5. Research Methodology

The aim of the research is to verify the possibility of functionally expanding the space observed by the robot system while maintaining the accuracy offered by advanced commercial 3D vision systems operating in a limited space and integrated industrially on robotic stations. The limited space in the case of standard computer vision solutions used in robotic stations results from limitations related to the resolution and depth of field of vision systems. It is worth referring here to the parameters of the 3DV/400 vision system, which is part of the workstation. The 3DV/400 system itself provides detection and localization of objects in the measurement volume of W2 × D2 × H2 = 527 mm × 460 mm × 1145 mm (Figure 3 and Figure 4). The spatial digital awareness of the robot system presented at the beginning of the article is a new concept in the context of industrial robotics, introduced by the author. In the literature, we can find the following definitions relating to spatial awareness: “… Spatial awareness is a fascinating cognitive skill that plays a crucial role in how we interact with our environment. It consists in understanding the relationship between objects in space and our position in relation to them…” [54]. Spatial awareness is a design approach that brings the physical and digital realms together. The ultimate goal is to create an environment where virtual objects seamlessly interact with the physical world, erasing the boundaries between them. This integration allows users to engage with digital content in a way that feels completely natural and instinctive [55]. “… Awareness of the environment (i.e., alertness) can be a kind of reflection of the characteristics of the environment in the mind. One of the manifestations of consciousness understood in this way is the representation of objects perceived visually…” [56]

With regard to the robot system, it is defined by the author as the ability of the robot system and the sensor elements integrated with it to detect, identify, and locate—in the workspace—elements belonging to user-specified categories, while maintaining all the features of the robot system that enable its industrial use. In this case, as mentioned, the measuring space is limited only by the robot’s kinematics and the space being obscured by the manipulator design.

The methodology of the research included the following stages.

**Figure 9:**
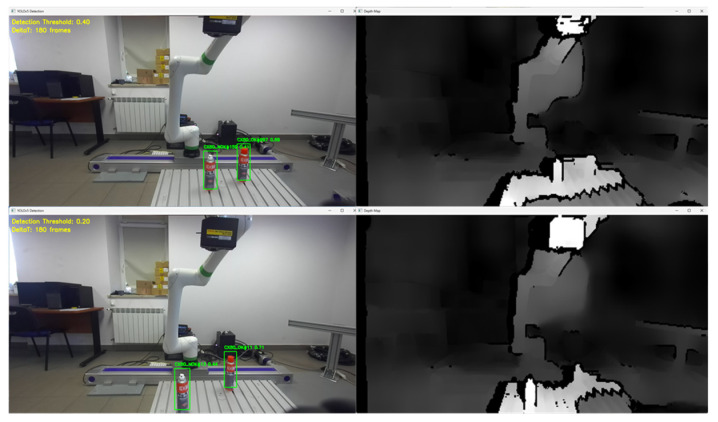
Examples of images showing the detection and location of CX80_OK and CX80_NOK objects in a 2D image, with the corresponding depth maps on the right side.

- Tools for recording and preparing training data were developed, followed by training for various network structures and YOLO models (YOLOv5 and YOLOv8). The use of algorithms from the YOLO family for object detection and identification allows for easy adaptation of the developed solution (change of weights obtained in the learning process) to the specific needs of customers.
- Research was conducted to verify the correctness of the learning process using the YOLOv5 and YOLOv8 algorithms and then implement the algorithm in the proprietary algorithm, allowing for the determination of 2D coordinates based on data recorded by the ZED 2i camera system.
- A key step was developing an application that allows reading data from the depth map recorded by the ZED 2i camera. To verify distance indications, proprietary software was used (the view of the data determined by the software is shown, e.g., in Figure 9) to verify the operation of the YOLOv8 algorithm and the algorithm for calculating 3D coordinates. The application enabled automatic reading of the 3D position of detected objects in the camera system and also allowed the user to indicate a point in the image (marked with a green cross in the place indicated by the user) and read the point’s coordinates. This allowed verification of the correctness of the application in determining the object position in 3D space.
- Indicating the position of objects in space for a robot system requires the transformation of data from the camera system to the robot’s coordinate system. For this purpose, a solution based on a common coordinate system of the ZED 2i robot and camera was used to precisely verify the position assigned by the developed algorithm.
- The next step was to develop a solution that transfers data from the computer system operating the ZED 2i camera to the robot’s system and moves the robot to a position that allows switching to the 3DV system. Using the FANUC RMI library, a Python application was developed that enables communication between the computer operating the ZED 2i camera and the CRX industrial robot controller.
- Verification of the developed algorithm—allowing expansion of the space in which the robot can operate based on data from the 3D vision system—was carried out as follows. Random points in the robot’s space were selected to verify the space at the near edge of the camera’s field of view and at the robot’s farthest reach, allowing the 3DV camera (integrated into the CRX robot’s wrist) to be positioned vertically above the object. For each position, the ZED 2i camera recorded 100 frames of the image and, on each frame, detected and located the objects. Based on the 100 frames for each object location, error calculations were performed to determine the errors resulting from the use of the YOLOv8 algorithm and the bounding box to determine the 3D position for each recorded image frame.
- For each selected point in the workspace, the average value of the 3D position was computed and the coordinates were used to move the 3DV camera, verifying correct coverage of the object’s field of view detected by the ZED 2i camera using the YOLOv8 algorithm. Errors were then determined between the position estimated by the vision system operating on ZED 2i data and the object’s actual position. Sample measurement points were placed both directly in front of the robot and within the maximum reach of the robot, taking into account the vertical positioning of the robot’s wrist to ensure correct operation of the 3DV system.

## 4. Results

The methods used to detect an object in space include the YoloV8 algorithm, which works on a 2D image. The algorithm has been trained to detect two strictly defined groups of objects (XC80_OK and XC80_NOK). The coordinates of the center of the detected object are used to read the distance of the object measured in the camera system. Based on this data, information about the position of the detected object in 3D space is transmitted via a proprietary application to the algorithm that controls the movement of the robotic arm via RMI. The TCP position relative to the actual position of the object is referred to as the positioning error at the detection and pre-positioning level. After moving the robot arm with the installed 3DV system camera to the position determined by the stereovision camera system, the vision process is started, which carries out a precise calculation of the position and orientation of the object in space. In this case, the errors are already minimal and are within the range resulting from the calibration errors of the vision system.

The results of the work carried out can be divided into three main parts: those related to the preparation of the stand and its launch; those related to the implementation of measurements at individual stages of the system’s operation; and those showing the benefits of implementing the developed solution. As already mentioned in the introduction, the aim of the developed solution was to increase digital awareness of the environment in the robot’s workspace. This was to allow the robot to carry out process operations in a wider area of its workspace.

### 4.1. Discussion of the Results of the Tests Carried Out

**Figure 10:**
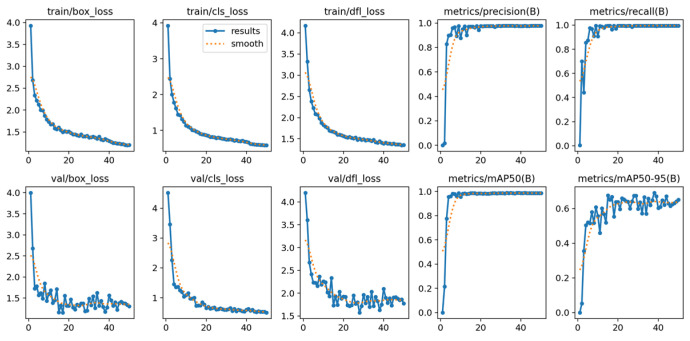
Results of learning the YoloV8 network to recognize objects in the form of CX80 belonging to classes OK (CX80 container with a lid) and NOK (CX80 container without a lid).

In order to detect objects in the workspace, and due to the available examples in the Stereolabs software, it was decided to prepare a training set for the Yolo algorithm. As already mentioned, at the research stage, training was carried out on the Yolov5s and Volov8n algorithms. In both cases, similar results were obtained, although, due to hardware limitations, training of the Yolov5 algorithm was carried out on batch8 (**batch** is a hyperparameter that defines the number of samples to work through before updating the internal model parameters) and Yolov8 on batch16 (Figure 10).

**Figure 11:**
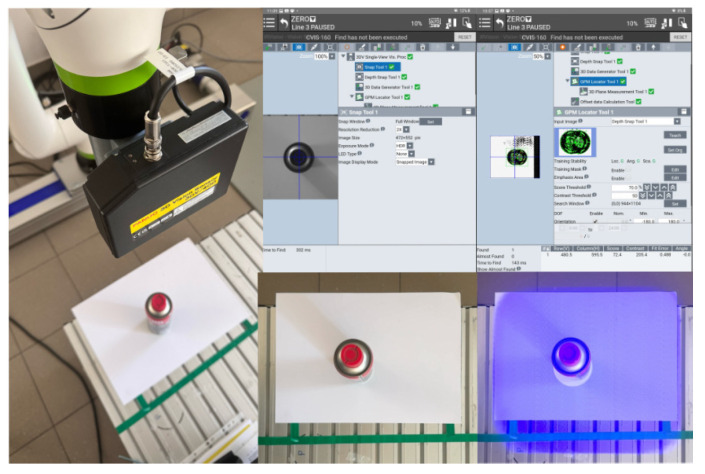
Configuration of the 3DV vision system with an image of the structured light system on the image surface.

**Figure 12:**
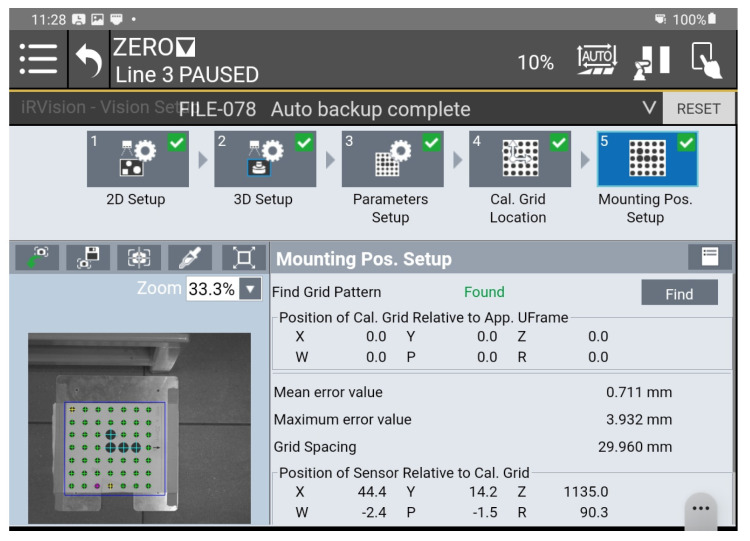
A view of the iRVision system panel showing the result of the vision system calibration.

**Figure 13:**
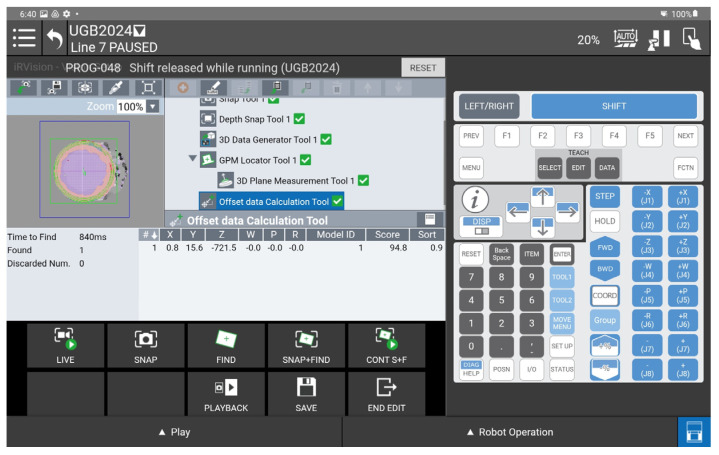
A view of the iRVision system panel showing the result of setting up the 3DV Vision Process tools.

Determination of the depth and position map based on information from the Yolov8 algorithm allowed for determining the position of the detected object, whose exact position in the ZED 2i camera system was determined using the robot and its TCP. (The actual position of the center of the CX80 object was determined (Figure 9); then the TCP was reached at the indicated point, and the TCP coordinates in the camera system were recorded (Figure 11, Figure 12 and Figure 13).)

**Figure 14:**
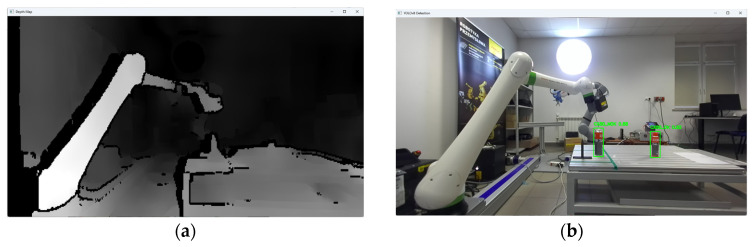
Depth image from the ZED2i camera (**a**) and image with the detected 2 objects CX80_OK and CX80_NOK (**b**) located on the table.

**Figure 15:**
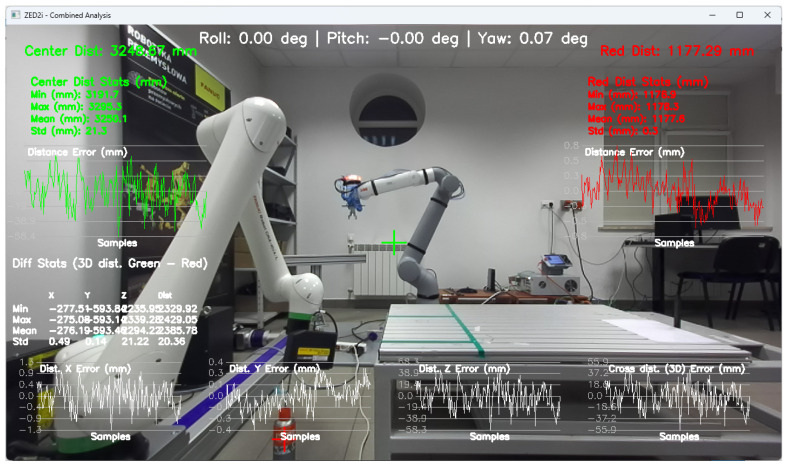
Image showing the view of the proprietary application for configuration and verification of the ZED2i camera, showing the distance measurement (result visible in red on the right) to the CX_80 object, located directly at the base of the robot (P01).

**Figure 16:**
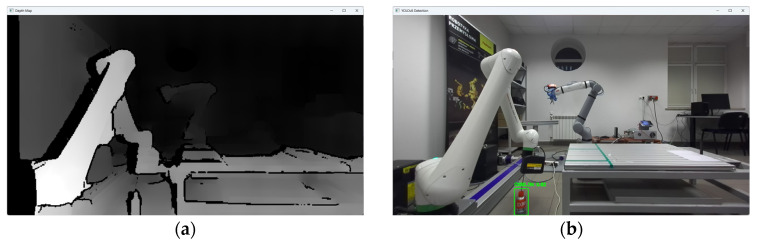
Depth image from the ZED2i camera (**a**) and image with the detected object CX80_OK (**b**) located directly at the base of the robot.

**Figure 17:**
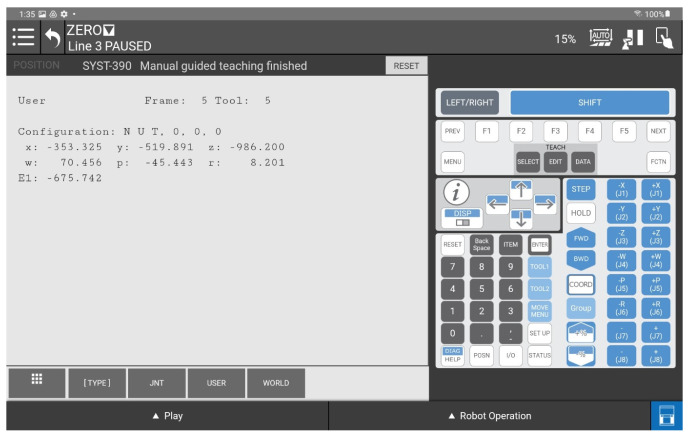
The position of an object outside the table, directly at the base of the robot, determined using TCP.

**Figure 18:**
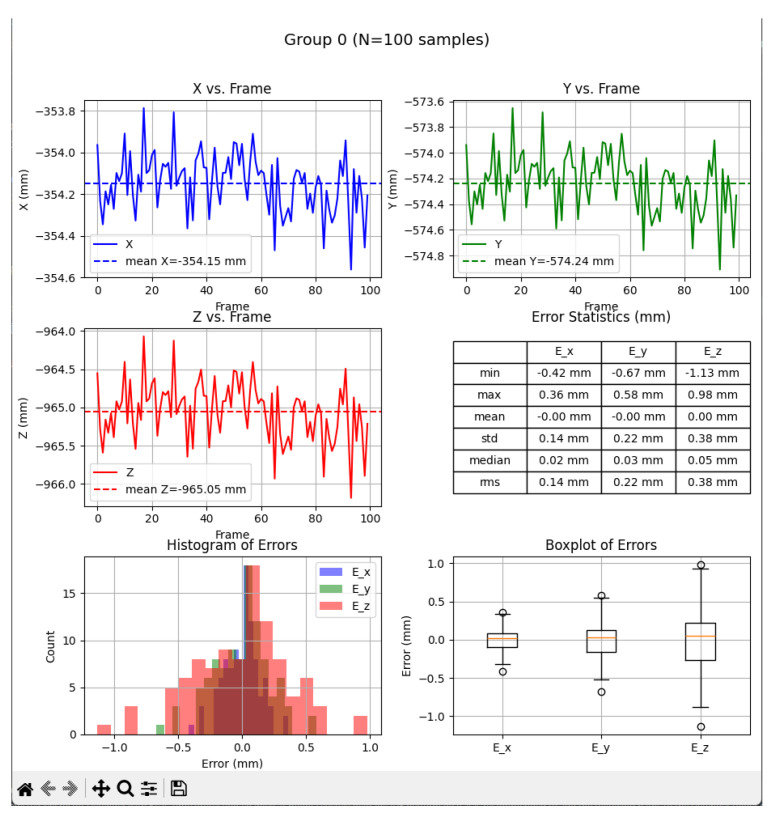
Analysis of position reading errors from the ZED2i camera system and the Yolov8 algorithm for the CX_80 object placed at the base of the robot.

The research was carried out, for example, at a dozen or so points in the space of the robot’s surroundings, in the field of view of the ZED2i vision system (Figure 14, Figure 15, Figure 16, Figure 17 and Figure 18). For each of the measuring points where the CX_80 tanks were placed, 100 image frames were collected, on which the position detection error analysis was performed. E_x and E_y errors result directly from specifying the center of the boundary of the detected objects. The E_z error, which corresponds to the distance of the object from the ZED2i camera coordinate system, is an error determined on the depth map based on the previously determined X and Y coordinates.

As shown in Table 4, the largest error occurred in the case of the Y coordinate, corresponding to the vertical axis. This is directly related to the fact that, during network training, YOLOV8 and the prepared bounding boxes did not always cover the entire object due to obscuration by obstacles and other objects. Another issue is the significant change in the object’s dimension due to the perspective and position of the object in relation to the axis of the camera layout. For this parameter (Y position), the impact is not critical because, from the point of view of the algorithm, the robot must still position itself above the detected object so that the object is in the 3DV vision system’s operating space. Errors in the Z axis are below 30 mm, which guarantees that the object is in the field of view of the 3DV system. The error in the distance to the object—as a parameter measured on the depth map—is the smallest and did not exceed 3 mm for any case. These results confirm the correctness of the assumptions regarding coarse positioning of the 3DV system with the help of the ZED 2i camera and the developed algorithm.

The results verifying the accepted hypothesis presented in this article were obtained on the basis of simulation and bench tests carried out by the author in laboratory conditions on a station equipped with a CRX robot with an IRVision 3DV vision system and a ZED2i stereovision camera from Stereolabs, working under the control of a Linux operating system with the use of Stereolabs libraries. The exchange of information between the robot system and the Stereolabs system was possible thanks to the use of the FANUC RMI software (R912) module, which provides the possibility of remote control of the robot’s motion based on data sent from an external computer.

### 4.2. Special Cases Worth Discussing

**Figure 19:**
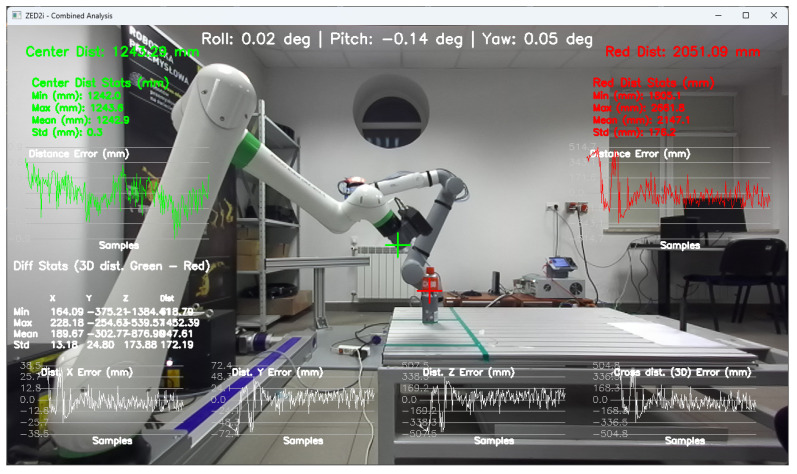
Verification of the distance to the object CX80_ON in the conditions of not using a screen in front of fluorescent lighting, using proprietary software.

**Figure 20:**
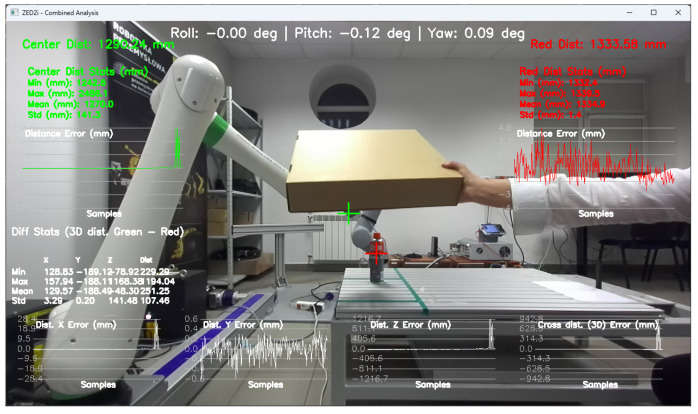
Verification of the distance to the object CX80_ON in the conditions of using a screen in front of fluorescent lighting, using proprietary software.

**Figure 21:**
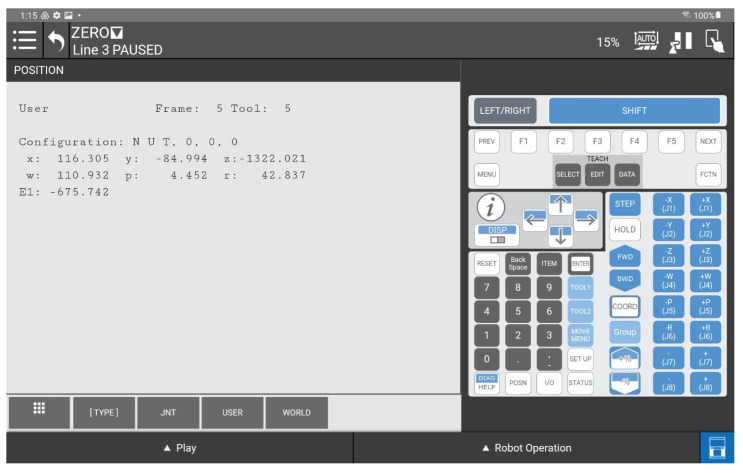
The physical position of the object CX80_OK measured using the robot’s TCP.

**Figure 22:**
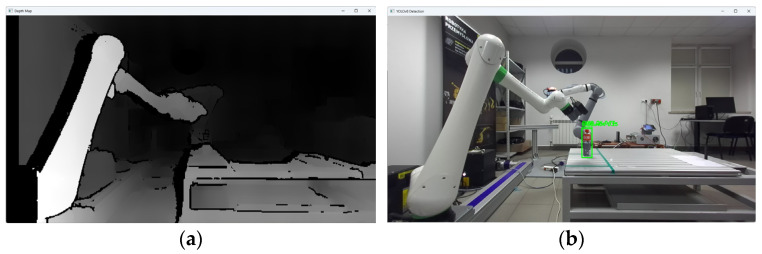
Depth image from the ZED2i camera (**a**) and image with the detected object CX80_OK, and (**b**) object located on the table, if the fluorescent lighting is not obscured.

**Figure 23:**
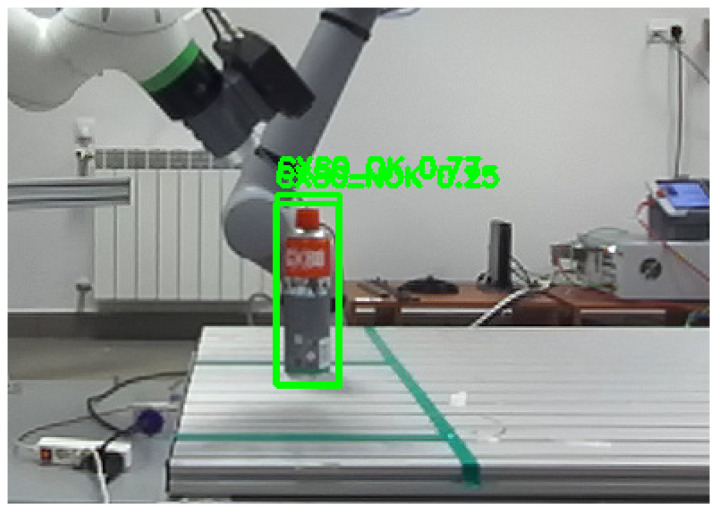
Duplicate detection of the same object as CX80_OK and CX80_NOK if the fluorescent lighting is not obscured.

During the research, occasional incorrect determinations of the distance to the object were noticed. Various causes of errors were considered, including an excessively large or small distance from the camera, obscuring by elements in the field of view in the immediate vicinity of the CX80, and background influence on the correct reading of the distance from the depth map. After a series of tests and trials, examining the position of the CX80 container in almost the entire field of view, the likely source of the error was identified as light reflections on the surface of the CX80 container, which could have come from the laboratory’s fluorescent lighting (Figure 19, Figure 20, Figure 21, Figure 22 and Figure 23).

After a more thorough analysis and use of the proprietary application applied for the research, one more detail was noticed. In the absence of an object obscuration, the Yolov8 application also generated errors by detecting two objects in the same image, one of which was CX80_OK and the other CX80_NOK. Unfortunately, the application itself could no longer correctly determine the distance or position of the object.

**Figure 24:**
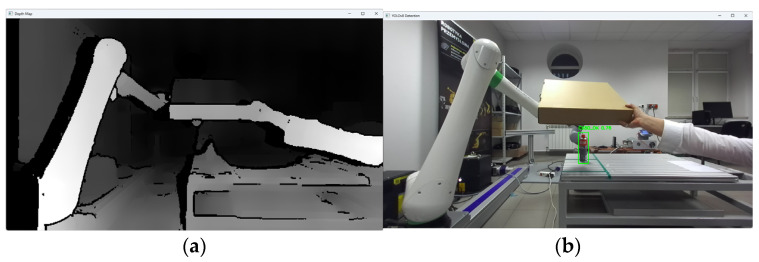
Depth image from the ZED2i camera (**a**) and image with the detected object CX80_OK (**b**) object located on the table, if the fluorescent lighting is obscured.

**Figure 25:**
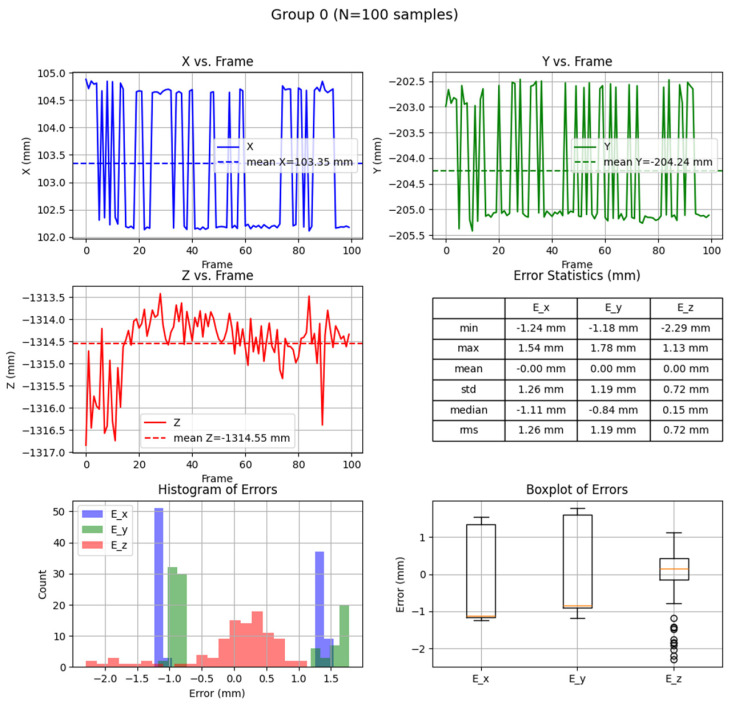
Analysis of position reading errors from the ZED2i camera system and Yolov8 algorithm for the CX_80 object located on the table, if the fluorescent lighting is obscured.

After covering the CX80 container, the correct operation of the application was observed, as well as the correct reading of the object coordinates calculated by the proprietary software (Figure 24 and Figure 25).

The results and observations presented above indicate the need to take into account the study of lighting conditions before commercial use of the developed solution. Of course, in the analyzed case, it is possible to consider the use of a camera with a lens equipped with a polarizing filter, or to analyze the possibility of extending the exposure time in order to avoid the impact of frequent fluorescent flashing on image recording.

## 5. Discussion

Combining the ZED2 and FANUC iRVision 3DV with the RMI library has the potential to significantly increase the flexibility and precision of machine vision in industrial robotics. This is particularly useful for tasks that require a broad perception of the environment and precise manipulation, although it comes with integration and cost challenges. On the one hand, it allows for a very wide field of view of the robot’s surroundings, and on the other hand, it does not degrade the accuracy offered by professional 3D vision systems that are normally dedicated to working with robotic stations. Similar tasks (searching for objects in the workspace using the 3DV system) would be possible, but they would require multiple manipulator movements and activation of the vision system. In this article, the use of the RMI module has been treated in a very limited way relative to the possibilities offered by this module. However, the focus was on verifying the possibility of integrating individual system elements and checking whether the positioning accuracy determined on the basis of ZED 2i would allow the keypad with the vision system to be directed to such a point of space where it would be possible to effectively detect the learned object of the iRVision system process. Of course, the use of Yolo algorithms and the ZED2i camera allows very broad scenarios for such a robotic station. In this example, Yolo was trained to recognize two types of CX80 cases: CX80_OK (a container with a lid) and CX80_NOK (a container without a lid). It is possible to teach the Yolo algorithm a much larger group of objects and to adjust the algorithm running on an external computer so that the robot control using the RMI library allows for triggering the procedure of automatic gripper change, and then handling specific objects. Compared to the research presented in the literature, the developed solution indicates a new point of view, especially in the field of industrial robots. It does not focus on improving positioning accuracy, but on expanding knowledge about the space defined in the article as digital operational awareness. Ultimately, this awareness can be used not only to detect process objects in the workspace, but also to detect situations that may lead to emergency situations or even dangerous situations for humans.

It is worth noting here two more important elements that will be analyzed at later stages of work. The solution presented in the article was based on the use of a single ZED 2i camera. This involves certain limitations related to the obstruction of the field of view by obstacles in the camera’s field of view and by the robot itself, which obscures part of the workspace. In future articles, the author intends to use the NVIDIA^®^ Jetson™ Orin NX (JAPAN FANUC) 16 GB module, which allows two stereo cameras to be connected, to verify the possibility of covering the full workspace with the vision system and to test the accuracy of one-camera and two-camera layouts. As in the presented example, it will be necessary to use the RMI module to control the movement of the FANUC CRX-10L robot. However, two variants of using this solution will be considered. In the first, the manipulator motion with the transfer of the camera of the 3DV system will be carried out directly from the algorithm on an external computer. In the second, the call of subprograms in the robot controller will be tested based on the data written in the position registers. In this case, the aim is to verify the effectiveness of robot control in two different algorithm variants. The series of three articles describing the solution presented here will be completed by investigating the possibility of dynamic robot-trajectory planning for a system using two ZED 2i stereo cameras, with the main trajectory planning algorithm generated on an external NVIDIA Jetson™ Orin NX computing^®^ unit.

Combining the ZED 2 and FANUC 3D Area Sensor using the RMI library could significantly increase the flexibility and precision of machine vision systems in industrial robotics. This is particularly useful for tasks that require a broad perception of the environment and precise manipulation, although it comes with integration and cost challenges.

When it comes to perceiving the environment and reacting to objects in space, the developed solution is most easily compared to solutions used in humanoid robots. In relation to those mentioned and as shown in Table 5, the proposed and verified solution is characterized by a much smaller number of cameras for observing the working area. Due to the location of the cameras (in the target version of the commercial solution, two cameras will be used outside the station in order to eliminate dead spaces resulting from the robot being obscured by the manipulator), the presented solution allows for continuous observation of the robot itself. The use of a stereovision camera with structured light allows for high precision in determining the poses of detected objects.

In terms of cost, the use of Stereolabs’ current ZED 2i cameras on the market, combined with a dedicated NVIDIA processor-based PC, provides a fully functional system (including an upgrade with two ZED 2i cameras) at a price below the price of the FANUC iRVision 3DV system alone. The obtained functionality—taking into account the possibility of easy modification of detected objects using the YOLO algorithm and observation of the full working space on the robotic station—can be used not only for the detection of process objects but also for the detection of people (not replacing the safety system due to the lack of appropriate safety certificates) in order to prevent activation of the station when it may be dangerous.

Of course, in addition to the ZED 2i and iRVision 3DV solutions, there are other 3D vision systems that can be used to achieve digital spatial awareness (Table 6).

By analyzing the parameters (Table 7), complementarity and the possibility of integration with the robot’s system of available systems, it is evident that the combination of ZED 2i and iRVision 3DV seems to be the optimal solution.

The main benefits of such a combination are obtaining a hierarchy of perception (from detection using AI to precision using structured light) and a pipeline (course-to-fine) that imitates the human cognitive approach. The developed and tested connection allows for better management of the robot’s movement. ZED2i communicates to the robot “where something is”. FANUC 3DV provides metric positions and orientations for trajectory planning. This makes it possible for both systems to operate in quasi-parallel—saving cycle time. Scalability and openness are also ensured. ZED2i works with ROS, NVIDIA Jetson, C++/Python SDK, powering multiple robots at the same time. iRVision is a native system, independent of external PCs—stable and industrial. The solution is also open to the possibility of adding more ZED2i cameras and thus eliminating the main weakness of the tested solution, which is that the robotic manipulator obscures the field of view of the ZED2i camera system.

## 6. Conclusions

The presented results confirm the correctness and practical usefulness of the developed solution. With the currently available commercial solutions offering the integration of vision systems with industrial robots, it is not possible to build such a broad digital awareness of the environment on a robotic station. After analyzing the literature, it is worth noting that articles from scientific journals have not yet presented a problem formulated in such a way, let alone a solution to the problem posed. Typically, the literature has focused on the integration of 2D and 3D sensors for a larger field of view or on the use of AI algorithms in industrial robotics. In both cases, these types of solutions are widely described for issues related to mobile robotics [67], as well as issues that allow for the optimization of the positioning process without taking into account the precision at the level presented in the article (cartons, etc.). The developed solution allows for maintaining the precision of the robotic system while extending its perception to almost the entire workplace, which is an important innovation that eliminates a significant problem occurring on robotic stations. It is also important that this solution is currently being implemented by one of the integrators dealing with robotic sorting, packaging and palletizing processes, and the use of the YOLO algorithm allows for a very simple adaptation of the algorithm to the changed assortment by changing the weights of the YOLO model.

In order to implement the developed solution on a real process station, additional environmental tests should be carried out to verify the impact of vibrations, dust, moisture or electromagnetic interference occurring at the station. However, it is worth mentioning here that the choice of the ZED2i camera solution provides immunity to some of the exposures that occur, as the camera itself and the available computing units offered by Stereolabs meet the immunity standards for equipment operating in demanding environmental conditions.

The features that distinguish the developed solution from other solutions available on robotic stations are as follows:

- Ensuring coverage of the full working area with detection and location of objects (excluding the space covered by the robotic manipulator).
- Ensuring the accuracy of positioning and orientation of the 3DV vision system throughout the entire space in which the integrated vision system can operate (excluding the space obscured by the robotic manipulator and taking into account the kinematic limitations related to the pose of the 3DV vision system installed on the robot’s wrist).
- Ensuring easy integration of the developed solution into the robot system.
- The ability to include the algorithm of communication and control of the robot, detection of objects belonging to different classes with simultaneous determination of their position in space, all in one cycle.
- Integration of two vision systems within one control system of the robotic station.
- Easy adaptation to new patterns.
- Ensuring the ability to easily adapt the algorithm to new products.
- Ensuring consistency of the solution with the robot control system.

Innovation, as defined by innovation leaders such as the consulting firm McKinsey and IDEO, encompasses several key characteristics:

- The ability to develop, deliver, and scale new products, services, processes, and business models for customers.
- The process of implementing an idea from the beginning to its implementation.
- Knowledge of when and how to use methods that allow for the development of new ideas.

We can also refer to the following definition contained in the standards: Innovation is the practical implementation of ideas that result in the introduction of new goods or services or improvement in offering goods or services. ISO TC 279 in the standard ISO 56000:2020. Others have different definitions; a common element in the definitions is a focus on newness, improvement, and the spread of ideas or technologies.

It can be assumed that, against the background of the presented definition and the results obtained, it can be unequivocally stated that the solution developed, tested, and presented in the article fits into the definition of an innovative solution. None of the known industrial or even research solutions can match the parameters of the solution described in the article, and the resulting fusion creates new opportunities for the use of data at robotic workstations.

The features that speak for the innovativeness of the developed solution are as follows:

- Coarse-to-fine architecture—Wide object detection (ZED 2i + YOLOv8) and local, fast positioning by 3D Area Sensor in a single motion sequence.
- Practical fusion of sensors on the robot—combining long-range stereovision with the designed short-range light increases the EFFECTIVENESS of the grip while maintaining awareness of the context (obstacles, scene changes).
- Semantic spatial awareness—YOLOv8 detections are converted into semantic points/poses in 3D, and then into robot movements using RMI, i.e., the robot “understands what and where it is”, not just “where something is”.
- Field of view expansion without loss of precision—ZED 2i gives FOV up to ~120° and 0.3–20 m, while the final precision is provided by the 3D Area Sensor (fast acquisition, high 3D resolution).
- Modularity and scalability—AI computing on PC (Python) + communication with the controller allows exchanging models, cameras and logic without changing the robot controller.
- Tough—Industrial enclosures and protection ratings (ZED 2i and IP66, FANUC 3D Area Sensor IP67) allow safely transferring a concept to a real target and using it in real processes.
- Short path from detection to motion—the combination of RMI/sockets + iRVision shortens the chain: detection → transformation → motion (less integration than in completely external systems).
- Ready to upgrade with 3D safety—One’s “awareness” is not safety-rated, but is easy to supplement with a certified 3D sensor (e.g., SICK), which opens the way to advanced collaborative goals.

Further work is currently underway to eliminate blind spots (use of an additional set of cameras) and to increase the precision and optimization of the algorithm in terms of performance. In addition, the algorithm will be enhanced with dynamic trajectory correction functions to eliminate potential collisions. Due to the applied nature of the developed solutions, it is important to maintain the features that allow them to be used on real robotic stations.

## Figures and Tables

**Table 1:** Parameters of Stereolabs cameras.

	ZED X	ZED X MINI	ZED 2I		ZED MINI
Focal length	2.2 mm	2.2 mm	2.1 mm	4 mm	1.4 mm
Default range	from 0.5 m to 20 m	from 0.2 m to 10 m	from 0.5 m to 20 m	from 1.5 m to 35 m	from 0.2 m to 10 m
Minimum Scope	0.3 m	0.1 m	0.3 m	1.5 m	0.1 m
Maximum range	20 m *	8 m *	20 m *	35 m *	15 m *

* In favorable conditions.

**Table 2:** Object detection performance comparison (YOLOv8 vs. YOLOv5) [30].

Model Size	Yolov5	Yolov8	Difference
Nano	28	37.3	+33.21%
Small	37.4	44.9	+20.05%
Medium	45.4	50.2	+10.57%
Large	49	52.9	+7.96%
Xtra Large	50.7	53.9	+6.31%

Image size = 640.

**Table 3:** Parameters of 3DV iRVision models.

	3DV/70	3DV/200	3DV/400	3DV/600	3DV/1600
Maximum 2D- and 3D- resolution [pixel]	870 × 950	1060 × 950	1104 × 950	1104 × 950	2208 × 1920
Acquisition time (w/o exposure time) [ms]	70	70	70	70	280
Measurement area W1 × D1 @ H1 [mm]	56 × 70 @167	124 × 124 @302	268 × 262 @645	576 × 499 @1245	1299 × 1223 @1448
Measurement area W2 × D2 @ H2 [mm]	83 × 92 @223	219 × 199 @492	527 × 460 @1145	804 × 697 @1745	3258 × 2843 @3448

**Table 4:** Examples of position measurement results (P 01–P 05) obtained for ZED2i in relation to the actual position of the object, measured with the use of a robot tool.

Position	P 01	P 02	P 03	P 04	P 05
Zed2 X (mm)	−354.15	−732.2	104.75	−22.09	−43.28
Zed2 Y (mm)	−574.24	−508.34	−205.25	−202.8	−205.54
Zed2 Z (mm)	−964.05	−2239.81	−1315.19	−1591.28	−1018
CRX TCP X (mm)	−353.325	−698.952	116.305	−3.793	−29.425
CRX TCP Y (mm)	−519.891	−383.976	−84.994	−84.993	−84.994
CRX TCP Z (mm)	−986.2	−2211.19	−1322.02	−1603.97	−1039.49
Error X (mm)	0.825	33.248	11.555	18.297	13.855
Error Y (mm)	54.349	124.364	120.256	117.807	120.546
Error Z (mm)	−21.15	28.623	−6.831	−12.693	−21.492
Error Z (%)	−2.29	1.28	−0.52	−0.79	−2.11

**Table 5:** Comparison of the developed solution and the solutions used in humanoid robots recording a digital image of the space in which they operate [57].

	Tesla Optimus	Boston Dynamics Atlas	Agility Robotics Digit	Figure AI Figure 02	Solution Developed
Total CMOS sensor number	8	13	5	9	2 (3 in future)
Camera Location	Head x4, Chest x2, Arms x2	Head x3, Chest x2, Wrists x4, Waist x4	Head x2, Chest x1, Arms x2	Head x3, Chest x2, Arms x4	Outside working area x1 (2 in future), Wrist x1
Camera Types	Wide-angle, Fisheye, Stereo cameras	ToF Cameras, Micro Cameras, RGB Cameras	Micro Cameras, Stereo cameras	ToF Cameras, RGB Cameras	Stereo Cameras (camera on Wrist with structured light)
Maximum Resolution	1920 × 1080	3840 × 2160	1920 × 1080	2560 × 1440	4416 × 1242
Chipset	Tesla FSD	NVIDIA	NVIDIA AGX	Proprietary Chipset	NVIDIA

**Table 6:** Comparison of the parameters of 3D vision systems that can be integrated with industrial robots [57,58,59,60,61,62,63,64,65,66].

System	Technology	Acquisition Time	FOV	Range (Min/Max)	Resolution	Accuracy/Precision	Time Efficiency	Use	Robot Integration
Cognex InSight L38 [53]	Laser line + embedded AI	<1 s (profile scan)	Profile Line (Variable)	Close (up to tens of mm)	Sub-mm (profile)	Sub-mm, μ scale	Fast—inline	Surface Inspection, Defects	Good (IO, Cognex tools)
Cognex InSight 3DL4000 [54]	Laser profiling (area-scan) with AI	Up to 5 kHz (Profile Linear Speed)	55 × 95 mm to 745 mm (depending on model)	~90–745 mm	960–1920 points/profiles	Z: 0.5–2 μm, X: ~28–99 μm	Very fast (line)	Robot guidance, profile, QA	Excellent (dedicated to robots)
FANUC iRVision 3DV (3DV/400) [55]	Structured light (IR single pattern)	~0.1–0.2 s/single measurement	268 × 262 mm @500 mm	~0.3–3 m	1104 × 950	~±0.5 mm; μm internally	Medium (~5–10 Hz)	Bin picking, positioning	Direct from FANUC
Intel RealSense D455 [56]	Stereo IR (Active IR with Projector)	up to 90 fps	87° × 58°	0.6–6 m	1280 × 720	<2% in the range of 2–4 m	High (do 90 Hz)	Mobile robots, prototypes	Open (ROS, SDK, no native integration)
Keyence VGR series [57]	Structured light + multi-camera	~136 images/session	~500 × 500 mm	0.3–1 m (Konfigurowalne)	~2 MP (Foldable Mesh)	~100–200 µm	Medium (~2–3 fps)	Depalletizing, picking	Good (with Keyence robot path planner)
Keyence XT (XT-024) [58]	Structured light with telecentric lens	~0.6 s	~60 × 60 mm	50–150 mm	9.44 MP + RGB	±10–20 μm, repeatability 0.5–1 μm	Low (~1.5–2 fps)	Micro-measurements, 3D inspection	Poor (mostly measuring, not guidance)
Photoneo MotionCam3D [59]	Structured light + snapshot CMOS	~20–50 ms (from 50 fps)	up to 1 m^2^ @ 1 m	0.2–2.0 m	from 3 MP (CMOS snapshot)	0.1–0.3 mm	Very high	Dynamic facilities, industry	Good (ROS, API)
Stereolabs ZED 2i [60]	RGB stereo + AI depth	15–100 FPS (depending on mode)	120° diagonal (~110° H)	0.2–20 m	2208 × 1242 (depth)	~1–3% of distance	High (30–100 fps)	Digital twins, location	Open (ROS, Jetson, SDK)
Zivid 2 + R-series (MR60/MR130) [61]	Structured light + HDR	MR: 150 ms, LR: 50 ms	MR60: 570 × 460 mm @600 mm	0.35–1.3 m (MR60/MR130)	5 MP	~80–210 μm	Medium (~7–20 fps)	Robots, automation, transparent objects	Excellent (ROS, SDK, Calibration Tools)

**Table 7:** Combining ZED2i and iRVision 3DV parameters to build digital spatial awareness.

	ZED2i	FANUC iRVision 3DV	Functional Sum
Range	0.2–20 m (ultra-wide)	0.3–3 m (depending on model)	Excellent task separation: wide detection + precise reading
Field of View (FOV)	120° (HD Stereo, AI Depth)	narrowed, object-oriented	ZED2i as a “radar”, 3DV as a “scalpel”
Spatial resolution	Medium; AI depth, RGB-D	μm precision + FANUC positioning	The result: first detection, then precise coordination
Environmental resilience	IP66, Integrated IMU, Polarity	IP65+, industry standards	Suitable for production halls and mobile robots
Robot integration	Via RMI (Robot Middleware Interface) or ROS	Direct integration into the FANUC controller	Robot movement possible immediately after location
Time efficiency	Detection of multiple objects simultaneously (100 Hz)	Precise triangulation of objects	The first system searches, the second confirms

## Data Availability

The Dataset is available on request from the author.

## Abbreviations

The following abbreviations are used in this manuscript:

**3DAS**	3D Area Sensor—version of 3D iRVision FANUC system
**3DL**	3D Laser—version of 3D iRVision FANUC system
**3DV**	3D Vision—version of 3D iRVision FANUC system
**AI**	Artificial intelligence
**EinH**	Eye-in-Hand
**EtoH**	Eye-to-Hand
**LIDAR**	Light Detection and Ranging
**RMI**	Remote Motion Interface
**SLAM**	Simultaneous Localization and Mapping
**TCP**	Tool Center Point
**ToF**	Time of Flight

## References

[1] Kaczmarek W., Panasiuk J. (2017). Robotization of Production Processes Introduction.

[2] Chen X., Guhl J. (2018). Industrial Robot Control with Object Recognition Based on Deep Learning. Procedia CIRP.

[3] Panasiuk J., Kaczmarek W. (2013). Wykorzystanie Systemu Wizyjnego Na Stanowiskach Zrobotyzowanych. Mechanik.

[4] Arents J., Greitans M. (2022). Smart Industrial Robot Control Trends, Challenges and Opportunities Within Manufacturing. Appl. Sci..

[5] Szabo R., Ricman R.S. (2023). Robotic Arm Position Computing Method in the 2D and 3D Spaces. Actuators.

[6] Ding Y., Hua L., Li S. (2022). Research on Computer Vision Enhancement in Intelligent Robot Based on Machine Learning and Deep Learning. Neural Comput. Appl..

[7] Hsu T.S., Wang T.C. (2015). An Improvement Stereo Vision Images Processing for Object Distance Measurement. Int. J. Autom. Smart Technol..

[8] Han P., Duan F., Li J., Wu L., Wang X. (2023). A Method for Improving Absolute Positioning Accuracy of Industrial Robot in Entire Workspace Domain Based on Stereo Vision. Opt. Precis. Eng..

[9] Wang Z., Jiao B., Xu L. Visual Object Detection: A Review. Proceedings of the Chinese Control Conference (CCC).

[10] Cheng F., Chen X. Integration of 3D Stereo Vision Measurements in Industrial Robot Applications. Proceedings of the 2008 IAJC-IJME International Conference.

[11] Grudziński M., Marchewka Ł. (2019). A Stereovision System for Three-Dimensional Measurements of Machines. Sci. J. Marit. Univ. Szczec..

[12] Yang W., Qin L., Hu X., Zhao D. (2023). Indoor Visible-Light 3D Positioning System Based on GRU Neural Network. Photonics.

[13] Kaczmarek W., Panasiuk J. (2017). Sensors and Sensory Systems of Industrial Robots.

[14] Marrón-Romera M., García J.C., Sotelo M.A., Pizarro D., Mazo M., Cañas J.M., Losada C., Marcos Á. (2010). Stereo Vision Tracking of Multiple Objects in Complex Indoor Environments. Sensors.

[15] Luo Y., Li S., Li D. (2020). Intelligent Perception System of Robot Visual Servo for Complex Industrial Environment. Sensors.

[16] Arokia Priya R., Patil A.V., Bhende M., Thakare A., Wagh S. (2022). Object Detection by Stereo Vision Images.

[17] Brecht F., Bart W., Luc B., Jose Ramon L.G., Ferrero T. (2010). Industrial Robot Manipulator Guarding Using Artificial Vision. Robot Vision.

[18] Luo J., Zhou X., Zeng C., Jiang Y., Qi W., Xiang K., Pang M., Tang B. (2024). Robotics Perception and Control: Key Technologies and Applications. Micromachines.

[19] Raj R., Kos A. (2025). An Extensive Study of Convolutional Neural Networks: Applications in Computer Vision for Improved Robotics Perceptions. Sensors.

[20] Gonçalves A., Pereira T., Lopes D., Cunha F., Lopes F., Coutinho F., Barreiros J., Durães J., Santos P., Simões F. (2025). Enhancing Nut-Tightening Processes in the Automotive Industry: Integration of 3D Vision Systems with Collaborative Robots. Automation.

[21] Valero S., Martinez J.C., Montes A.M., Marín C., Bolaños R., Álvarez D. (2025). Machine Vision-Assisted Design of End Effector Pose in Robotic Mixed Depalletizing of Heterogeneous Cargo. Sensors.

[22] El Ghazouali S., Mhirit Y., Oukhrid A., Michelucci U., Nouira H. (2024). FusionVision: A Comprehensive Approach of 3D Object Reconstruction and Segmentation from RGB-D Cameras Using YOLO and Fast Segment Anything. Sensors.

[23] Zhou Y., Rashid F.A.N., Mat Daud M., Hasan M.K., Chen W. (2025). Machine Learning-Based Computer Vision for Depth Camera-Based Physiotherapy Movement Assessment: A Systematic Review. Sensors.

[24] Tzampazaki M., Zografos C., Vrochidou E., Papakostas G.A. (2024). Machine Vision—Moving from Industry 4.0 to Industry 5.0. Appl. Sci..

[25] Zhang J., Wang Z., Lai J., Wang H. (2025). GPTArm: An Autonomous Task Planning Manipulator Grasping System Based on Vision–Language Models. Machines.

[26] Silva C.A.d.S., Paladini E.P. (2025). Smart Machine Vision System to Improve Decision-Making on the Assembly Line. Machines.

[27] Schorr L., Cobilean V., Mavikumbure H.S., Manic M., Hadimani R.L. (2025). Industrial workspace detection of a robotic arm using combined 2D and 3D vision processing. Int. J. Adv. Manuf. Technol..

[28] Liu Y., Cui X., Fan S., Wang Q., Liu Y., Sun Y., Wang G. (2024). Dynamic Validation of Calibration Accuracy and Structural Robustness of a Multi-Sensor Mobile Robot. Sensors.

[29] Ye F., Jia G., Wang Y., Chen X., Xi J. (2024). Kinematic and Joint Compliance Modeling Method to Improve Position Accuracy of a Robotic Vision System. Sensors.

[30] Burke I., Salzer S., Stein S., Olusanya T.O.O., Thiel O.F., Kockmann N. (2024). AI-Based Integrated Smart Process Sensor for Emulsion Control in Industrial Application. Processes.

[31] Xu J., Liu Q., Xu Y., Xiao R., Hou Z., Chen S. (2024). Review on the Application of the Attention Mechanism in Sensing Information Processing for Dynamic Welding Processes. J. Manuf. Mater. Process..

[32] Fatima Z., Zardari S., Tanveer M.H. (2024). Advancing Industrial Object Detection Through Domain Adaptation: A Solution for Industry 5.0. Actuators.

[33] Flandin G., Chaumette F., Marchand E. Eye-in-hand/Eye-to-hand Cooperation for Visual Servoing. Proceedings of the 2000 ICRA. Millennium Conference. IEEE International Conference on Robotics and Automation. Symposia Proceedings (Cat. No.00CH37065).

[34] Lippiello V., Siciliano B., Villani L. Eye-in-Hand/Eye-to-Hand Multi-Camera Visual Servoing. Proceedings of the 44th IEEE Conference on Decision and Control.

[35] Lippiello V., Siciliano B., Villani L. (2007). Position-Based Visual Servoing in Industrial Multirobot Cells Using a Hybrid Camera Configuration. IEEE Trans. Robot..

[36] Chang W.-C., Shao C.-K. Hybrid Eye-to-Hand and Eye-in-Hand Visual Servoing for Autonomous Robotic Manipulation. Proceedings of the SICE Annual Conference 2010.

[37] Cuevas-Velasquez H., Li N., Tylecek R., Saval-Calvo M., Fisher R.B. Hybrid Multi-camera Visual Servoing to Moving Target. Proceedings of the 2018 IEEE/RSJ International Conference on Intelligent Robots and Systems (IROS).

[38] AlBeladi A., Ripperger E., Hutchinson S., Krishnan G. (2022). Hybrid Eye-in-Hand/Eye-to-Hand Image-Based Visual Servoing for Soft Continuum Arms. IEEE Robot. Autom. Lett..

[39] Vincze M., Patten T., Park K., Bauer D. (2020). Learn, Detect, and Grasp Objects in Real-World Settings. Elektrotech. Inf..

[40] Amin F.M., Rezayati M., van de Venn H.W., Karimpour H. (2020). A Mixed-Perception Approach for Safe Human–Robot Collaboration in Industrial Automation. Sensors.

[41] Zhao C., Sun L., Stolkin R. (2020). Simultaneous Material Segmentation and 3D Reconstruction in Industrial Scenarios. Front. Robot. AI.

[42] Kaczmarek W., Panasiuk J., Borys S. (2017). Środowiska Programowania Robotów.

[43] Diao S., Chen X., Luo J. (2018). Development and Experimental Evaluation of a 3D Vision System for Grinding Robot. Sensors.

[44] Marny M., Hetmanczyk M.P. (2020). Configuration and Programming of the Fanuc Irvision Vision System for Applications in the Dynamic Environment of Manipulated Elements. Int. J. Mod. Manuf. Technol..

[45] Pally R.J., Samadi S. (2022). Application of Image Processing and Convolutional Neural Networks for Flood Image Classification and Semantic Segmentation. Environ. Model. Softw..

[46] Martínez-Corral M., Javidi B. (2018). Fundamentals of 3D Imaging and Displays: A Tutorial on Integral Imaging, Light-Field, and Plenoptic Systems. Adv. Opt. Photon.

[47] Michalos G., Makris S., Eytan A., Matthaiakis S., Chryssolouris G. (2012). Robot Path Correction Using Stereo Vision System. Procedia CIRP.

[48] Pestana D., Miranda P.R., Lopes J.D., Duarte R.P., Vestias M.P., Neto H.C., De Sousa J.T. (2021). A Full Featured Configurable Accelerator for Object Detection with YOLO. IEEE Access.

[49] de Souza J.P.C., Amorim A.M., Rocha L.F., Pinto V.H., Moreira A.P. (2022). Industrial Robot Programming by Demonstration Using Stereoscopic Vision and Inertial Sensing. Ind. Robot..

[50] Tadic V. (2023). Study on Automatic Electric Vehicle Charging Socket Detection Using ZED 2i Depth Sensor. Electronics.

[51] Geng J. (2011). Structured-Light 3D Surface Imaging: A Tutorial. Adv. Opt. Photonics.

[52] https://www.augmentedstartups.com/blog/yolov8-vs-yolov5-choosing-the-best-object-detection-model?srsltid=AfmBOopETe6qH_aDukxF9UG5HR9JbJ55jHvr1Bddoc9T4FWECxwvI_lE.

[53] (2020). RMI_Operator_Manual_[B-84184EN_2]–FANUC 2020.

[54] What Is Spatial Awareness? 7 October 2024 Claire Andersonno Comments Yet. https://focuskeeper.co/glossary/what-is-spatial-awareness.

[55] https://medium.com/design-bootcamp/spatial-awareness-with-visual-pro-creating-immersive-experiences-for-users-5821e9882f69.

[56] https://en.wikipedia.org/wiki/Consciousness.

[57] https://www.trendforce.com/news/2025/01/14/insight-analysis-of-humanoid-robot-vision-systems-and-opportunities-for-taiwanese-companies/.

[58] https://support.cognex.com/docs/is3d_2410/EN/L38_Manual.pdf.

[59] https://www.cognex.com/en-pl/products/machine-vision/3d-machine-vision-systems/in-sight-3d-l4000/specifications.

[60] https://www.fanuc.co.jp/en/product/robot/function/irvision.html.

[61] https://en.wikipedia.org/wiki/Intel_RealSense.

[62] https://www.keyence.com/products/vision/vision-sys/3d_vgr.

[63] https://www.keyence.com/products/vision/vision-sys/3d_vision.

[64] https://www.photoneo.com/products/motioncam-3d-l.

[65] https://www.stereolabs.com/en-pl/store/products/zed-2i.

[66] https://en.wikipedia.org/wiki/Zivid.

[67] Cherubin S., Kaczmarek W., Siwek M. (2024). YOLO object detection and classification using low-cost mobile Robot. Prz. Elektrotech.

